# Identification of Pathogenicity Loci in *Magnaporthe oryzae* Using GWAS with Neck Blast Phenotypic Data

**DOI:** 10.3390/genes13050916

**Published:** 2022-05-20

**Authors:** Nyein Nyein Aye Myint, Siripar Korinsak, Cattleya Chutteang, Kularb Laosatit, Burin Thunnom, Theerayut Toojinda, Jonaliza L. Siangliw

**Affiliations:** 1 Faculty of Agriculture at Kamphaeng Saen, Kasetsart University, Kamphaeng Saen, Nakhon Pathom 73140, Thailand; nyeinnyeinayemyintdar@gmail.com; 2 National Center for Genetic Engineering and Biotechnology (BIOTEC), National Science and Technology Development Agency (NSTDA), Thailand Science Park, Phahonyothin, Khlong Nueng, Khlong Luang, Pathum Thani 12120, Thailand; siripar.kor@biotec.or.th (S.K.); burin.thu@biotec.or.th (B.T.); theerayut@biotec.or.th (T.T.); 3 Department of Agronomy, Faculty of Agriculture at Kamphaeng Saen, Kasetsart University, Kamphaeng Saen, Nakhon Pathom 73140, Thailand; agrcyc@ku.ac.th (C.C.); fagrkal@ku.ac.th (K.L.)

**Keywords:** *Magnaporthae oryzae*, neck blast, SNP, GWAS, haplotype

## Abstract

*Magnaporthae oryzae (M. oryzae)* is the most destructive disease of rice worldwide. In this study, one hundred and two isolates of *M. oryzae* were collected from rice (*Oryzae sativa* L.) from 2001 to 2017, and six rice varieties with resistance genes *Pizt*, *Pish*, *Pik*, *Pib,* and *Pi2* were used in a genome-wide association study to identify pathogenicity loci in *M. oryzae*. Genome-wide association analysis was performed using 5338 single nucleotide polymorphism (SNPs) and phenotypic data of neck blast screening by TASSEL software together with haplotype block and SNP effect analysis. Twenty-seven significant SNPs were identified on chromosomes 1, 2, 3, 4, 5, 6, and 7. Many predicted genes (820 genes) were found in the target regions of six rice varieties. Most of these genes are described as putative uncharacterized proteins, however, some genes were reported related to virulence in *M. oryzae*. Moreover, this study revealed that *R* genes, *Pik*, *Pish*, and *Pi2*, were broad-spectrum resistant against neck blast disease caused by Thai blast isolate. Haplotype analysis revealed that the combination of the favorable alleles causing reduced virulence of isolates against IRBLz5-CA carrying *Pi2* gene contributes 69% of the phenotypic variation in pathogenicity. The target regions and information are useful to develop marker-specific genes to classify blast fungal isolates and select appropriate resistance genes for rice cultivation and improvement.

## 1. Introduction

Rice is an important food crop and is very susceptible to diseases like blast, which is a known threat to global food security. This disease alone is responsible for approximately 30% of global yield loss [1]. Rice blast disease caused by teleomorph *Magnaporthe oryzae* asexual stage (anamorph *Pyricularia oryzae* (*P. oryzae*) sexual stage) is the most destructive rice (*Oryza sativa* L.) disease worldwide. Leaf blast and neck blast are the two rice blast pathosystems caused by the same fungus [2]. Rice blast disease can affect all parts of the rice plant, such as leaf, collar, node, panicle, neck, and seed. Among them, neck blast disease is the most destructive stage and can occur without being preceded by leaf blast. Neck blast causes direct loss of yield due to poor grain filling resulting to entirely sterile panicles. For this reason, neck blast is the most serious phase of blast disease [3]. Rice blast pathogen has high genetic diversity and the ability to modify itself to control resistance in a rice variety by parasexual reproduction, causing difficulties in developing resistant varieties [4,5]. Understanding the structure of the blast population and its genetic diversity is important for disease management. The breeding of resistant cultivars is the favored solution to prevent or reduce yield loss due to rice blast epidemics. The gene-for-gene hypothesis is based on the observation that disease resistance in plants usually requires two complementary genes: an avirulence gene (*Avr*) in the pathogen and a matching resistance gene (*R*) in the host [6]. The *Avr* protein or effector molecule secreted by a pathogen can directly/indirectly trigger a specific molecule in the plant to initiate a resistance pathway [7]. Therefore, knowledge of *Avr* genes is crucial to understand the resistance process and the potential utility of resistance genes. Currently, more than 40 *Avr* genes have been identified in rice blast fungus and only 11 *Avr* genes have been cloned, such as *PWL1*, *PWL2*, *Avr*-*Piz*-*t*, *Avr*-*Pita*, *ACE1*, *Avr*-*Pia*, *Avr*-*Pib*, *Avr1*-*CO39*, *Avr*-*Pik*, *Avr*-*Pii*, *Avr*-*Pi9,* and *Avr*-*Pi54* [8]. Most *Avr* genes have been identified by QTL mapping approaches and are specific to leaf blast disease.

Genome-wide association studies can be a powerful tool to identify candidate alleles that control quantitative and qualitative traits in plants, microbes, and animals [9]. GWAS has recently been used to evaluate associations between genetic markers and blast resistance in rice [10]. It has been used to identify the *Xa43*(*t*) novel resistance gene for bacterial blight in rice [11] and resistance loci for sheath blight disease in rice [12]. It has also been successfully used to identify single nucleotide polymorphisms (SNPs) associated with virulence genes on chromosomes 1 that might be linked with *AvrPi*7, 2, 3, 4, and 7 in *M*. *oryzae* against leaf blast disease [13]. Pathogenicity of the fungus can be changed when fungus infects different growth stages of rice. The fungus showed avirulence at the vegetative stage (leaf blast), but virulence at the reproductive stage (neck blast) of rice variety Zhong 156, which contained resistance gene *Pi24* [14]. The rice variety Aichinokaori with *Pb1* gene was susceptible at a young stage and resistant at reproductive stage [15]. Therefore, pathogenicity or infection mechanism of fungi may involve different genes at each growth stage. At present, no work has yet confirmed that pathogenicity gene-conferred virulence to leaf and neck and panicle blast disease are controlled by the same corresponding genes.

In this study, we aimed to determine the response of six rice cultivars, IRBLzt-T, IRBLsh-S, IRBLk-K, IRBLb-B, IRBLz5-CA, and Lijiangxintuangheigu (LTH) (a universal susceptible control) against blast fungus and identify SNPs associated with pathogenicity genes in *M*. *oryzae* corresponding to resistance genes for neck blast disease in the six cultivars, by analyzing the association between genetic variation and disease response. This study will provide more information to understand the genetics controlling the virulence of the fungus and will help breeders to select the appropriate *R* genes to be used in breeding programs to improve varieties with broad spectrum and durable resistance to rice blast.

## 2. Materials and Methods

### 2.1. Fungal Population

A total of 102 isolates of *M*. *oryzae* collected from several locations in Thailand between 2001–2017 were used for whole genome re-sequencing and disease screening on rice (Table S6). These were isolated from infected tissues of different rice varieties/cultivars including leaf, collar, neck, panicle, and seeds by using a monoconidial isolation method.

### 2.2. Plant Materials

Six rice varieties were used for neck blast disease screening. Five rice varieties containing different blast resistance genes were developed by the International Rice Research Institute (IRRI) [16] including IRBLzt-T, IRBLsh-S, IRBLk-KA, IRBLb-B, and IRBLz5-CA, which confer rice blast disease resistance genes *Pizt*, *Pish*, *Pik*, *Pib,* and *Pi2*, respectively, and Lijiangxintuangheigu (LTH); the genetic background of developed rice IRBL was used as universal susceptible check for disease evaluation.

### 2.3. Whole Genome Re-Sequencing

Genomic DNA was extracted by using a high purity PCR template preparation kit (Roche, Germany) from mycelia that were cultured and collected [13]. Measurement of DNA quality and concentration was conducted by running on 1% agarose gel and NanoDrop (ND-8000; Thermo Scientific, Wilmington, DE, USA). The whole genome re-sequencing was performed on the next generation platform MGISEQ-200 (BGI, Shenzhen, China) with paired-end 100 bp and a coverage of 20×. Sequences of all Thai isolates was compared with the public reference sequence of *M*. *oryzae* isolates70-15, assembly version MG8, (release-51: http://fungi.ensembl.org/Magnaporthe_oryzae/nfo/Index accessed on 20 September 2019).

### 2.4. Assessment of Neck Blast Disease, Avirulence, and Data Analysis

Neck blast disease inoculation was performed under greenhouse condition. Blast isolates were cultured for seven days at 25 °C on rice polish agar (RPA) medium, (20 g rice polish, 20 g agar, and 2 g yeast extract powder for 1 L). The mycelia were scraped, and sporulation was induced under blacklight cabinet for two days. Then, clean water was added to collect the conidia. The suspension of conidia was adjusted to a concentration of 1 × 10^4^ conidia/mL. Inoculation was carried out by injecting 1 mL per panicle at the reproductive stage–when the panicle emerges 1 to 3 cm above the flag leaf– using a syringe. Three replications of 10 plants per replicate were inoculated. The disease was assessed at 14 days after inoculation using a scale of 0, 1, 3, 5, 7, and 9 (0 is highly resistant and 9 is highly susceptible) [17]. The disease score was used to identify isolates as avirulence or virulence (0 is avirulence and 9 is virulence). Statistical analyses of the above six traits were performed using R software [18]. 

### 2.5. Association Analysis of Avirulence Genes in M. oryzae

Whole genome re-sequencing was performed across all blast isolates by MGIseq200, and the sequences were then compared with the reference sequence–isolate 70-15–and SNPs were called to constitute the genotype data. A total of 310,530 SNPs were filtered using PLINK version 1.9, by eliminating genotypes with >10% missing data and rare SNPs with <5% minor allele frequencies (MAF). Subsequently, the genotype was pruned based on linkage disequilibrium. By using PLINK version 1.9, SNPs having r^2^ correlation (indep-pairwise) more than 0.3 in every 50 kb were filtered out. After filtering, linkage disequilibrium (LD) was analyzed in PLINK version 1.9. The association analysis was performed by TASSEL version 5.7.2 using 5338 selected SNPs and infection phenotypes on the six rice varieties. SNPs above a threshold of −log10 (P) ≥ 3.0 were considered significantly associated with the six traits.

### 2.6. Haplotype Analysis and Candidate Genes

From the GWAS results, significantly associated SNPs were observed for their linkage disequilibrium blocks and haplotypes. Haploview software was implemented with the solid spine method for defining blocks in each chromosome [19]. After blocks were identified, we used R version 4.0.3 to conduct a multiple linear regression [20] of SNPs, including the GWAS SNP and other SNPs within the block to see whether there was any effect or association of other SNPs within the block with the trait. Multiple iterations were performed until the significant SNPs were not changed. In case there were more than one SNP remaining in a block, the tree-based SNP-SNP interaction analysis [21] was performed subsequently. Moreover, if there were several blocks with significant SNPs in a trait, the SNPs were combined, and multiple regression was again performed followed by n-way interaction analysis of the remaining significant SNPs.

In identifying candidate genes, linkage disequilibrium (LD) decay was considered. LD decay was analyzed by PopLDdecay software [22], and the result was then used as a reference in observing candidate genes on public databases such as EnsemblFungi for *M. oryzae* (MG8) (https://fungi.ensembl.org accessed on 1 April 2021), UniProtKB (www.uniprot.org accessed on 26 November 2021), and PHI-base (www.phi-base.org accessed on 27 November 2021).

## 3. Results

### 3.1. Neck Blast Disease Evaluation

**Figure 1:**
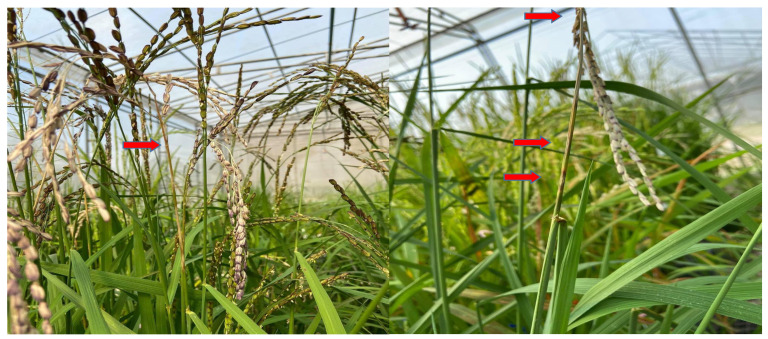
Neck and panicle inoculated with conidia suspension. (Red arrows show neck blast infection with complete white head panicle).

**Figure 2:**
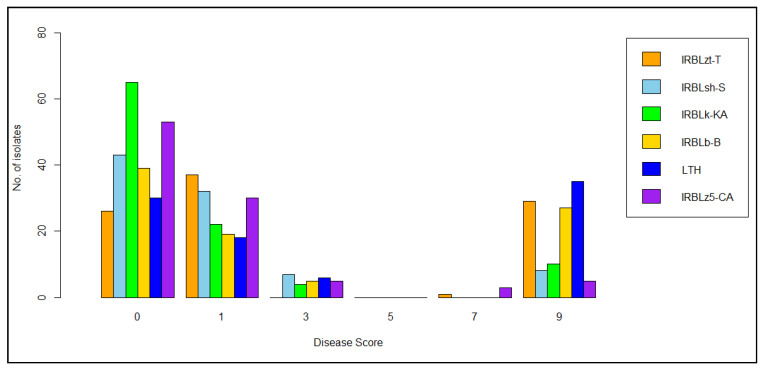
Frequency distribution of blast isolates in six rice varieties showing disease score of 91 isolates in IRBLzt-T, 90 isolates in IRBLsh-S, 96 isolates in IRBLk-KA, 85 isolates in IRBLb-B, 84 isolates in LTH, and 91 isolates in IRBLz5-CA.

Infected neck and panicle with completely sterile panicles are shown in Figure 1. All tested cultivars were affected by neck blast disease under greenhouse conditions. A histogram of disease scores against each rice cultivar shows a binomial distribution (Figure 2). Adding together the resistance reactions (HR, R, and MR in Table S1), IRBLsh-S (with resistance gene *Pish*), IRBLk-KA (*Pik*) and IRBLz5-CA (*Pi2*) were the top three broad-spectrum resistant (BSR) rice with levels of over 90%, while LTH had the lowest BSR at 60.71% (MS, S, and HS in Table S1) against Thai blast isolates.

The isolates’ disease scores were different across all six rice cultivars (Table S2). Isolate virulence was assessed, and results reveal that 31.4% of isolates were unable to cause blast disease across all six rice varieties. About 42% of the isolates were able to cause disease in one of the six rice varieties, while 19% and 7.8% of the isolates caused disease in two or three varieties, respectively. Only one isolate, TRG2, was found to be the most virulent, and could infect four out of six varieties.

### 3.2. Linkage Disequilibrium Decay

**Figure 3:**
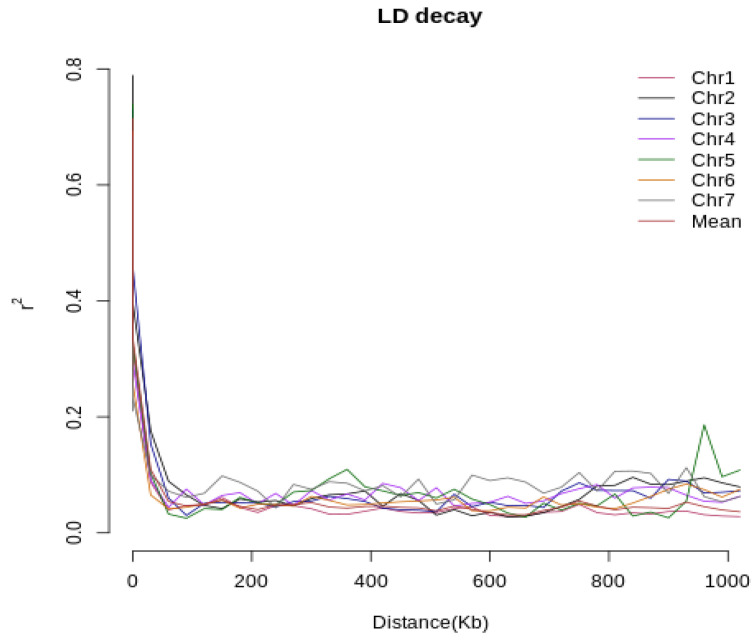
Linkage disequilibrium decay in blast isolates genome.

Linkage disequilibrium of the blast fungal isolate population reduced dramatically from 1 to 0.1 (Figure 3). Chromosomes 1 and 6 had the lowest r^2^ at 0.0874 and 0.0653, respectively (Table S3). Taking the average across all chromosomes, decay reached 0.1 at less than 50 kb distance. Following the linkage disequilibrium in rice, which commonly uses a cut-off threshold of 0.2 [22,23,24], the LD of isolates dropped down by half at less than 50 kb, resulting in a LD block of 30 kb less than average. 

### 3.3. Population Structure

**Figure 4:**
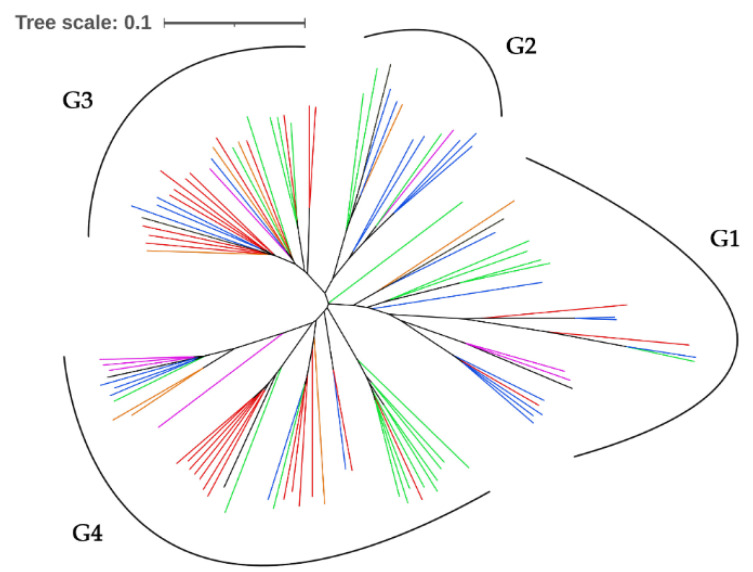
Principal component analysis of unrooted neighboring-joining clustering tree. Note: northeast—red; north—green; central—blue; south—purple; east—orange; west—black.

A total of 5338 SNPs identified from 102 *M*. *oryzae* isolates were analyzed using TASSEL version 5 for population structure. Principal component analysis was performed to determine the population structure or the familial relatedness of 102 blast isolates using the unrooted Neighbor-Joining clustering. Analyses indicate the distribution of different regions in Thailand from which the isolates were obtained (30 from northeast, 25 from central, 26 from north, 10 from south, 8 from east, and 3 from west). The population structure of *M*. *oryzae* was divided into four groups (G1, G2, G3, and G4) (Figure 4). Most of the northeast isolates were found grouped in G3 and G4. The isolates coming from the central part of Thailand are mostly found in G1 and G2, while G4 also contain isolates from southern Thailand. The remaining isolates belonging to the different regions such as from the north, south, east, and west of Thailand were found distributed within G1, G2, G3, and G4. Based on the disease reactions (Table S2), IRBLk-KA, IRBLsh-S, and IRBLz5-CA were found the most resistant against four groups of *M*. *oryzae* isolates. The susceptible reactions when grouped did not exceed 10% in IRBLk-KA, were less than 18% in IRBLsh-S, and less than 10% in IRBLz5-CA in any of the four groups of *M*. *oryzae* isolates. More than 50% of isolates coming from the east and west of Thailand were able to cause disease in IRBLb-B.

### 3.4. GWAS Analysis and Identification of Candidate Genes

**Figure 5:**
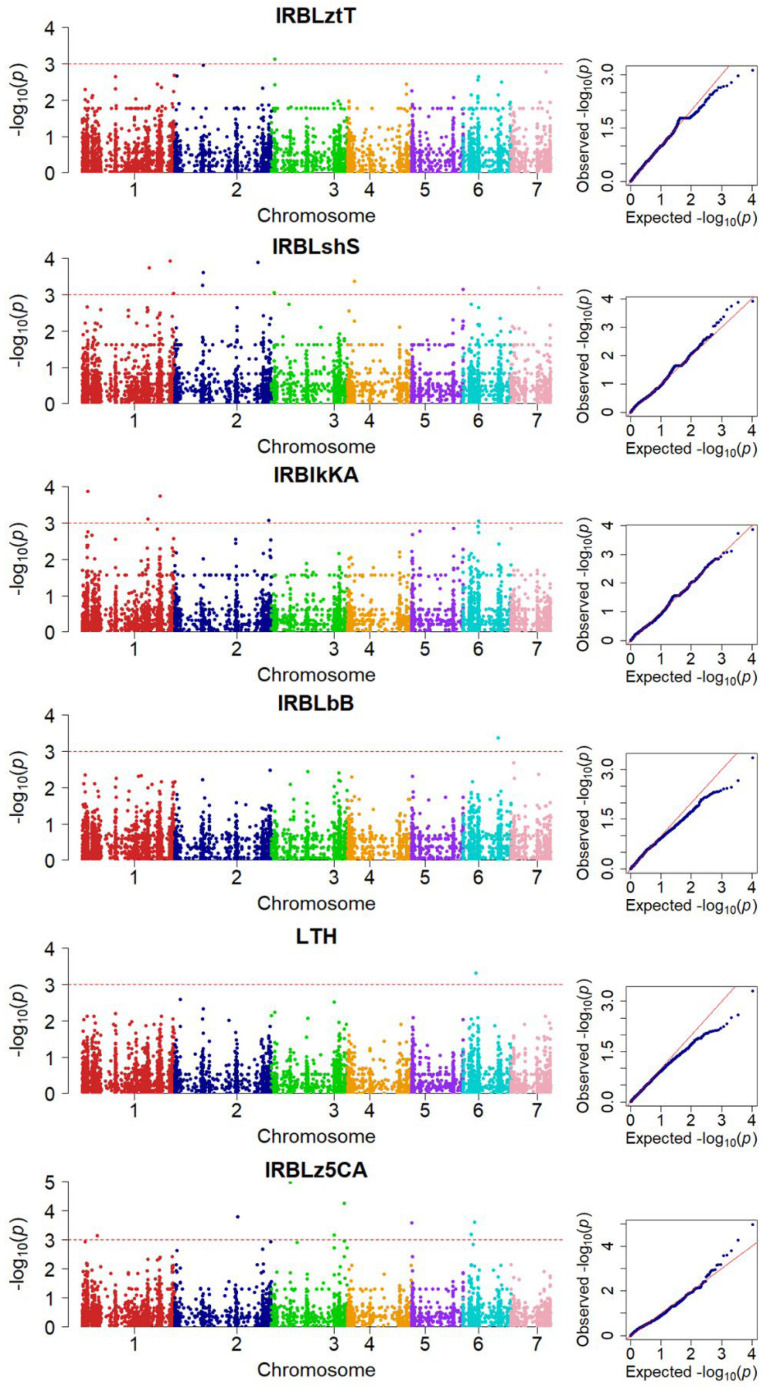
The Manhattan plots and Q−Q plots of genome-wide association study for six rice varieties inoculated with rice blast fungi using mixed linear model, 85 isolates in IRBLb-B, 96 isolates in IRBLk-KA, 90 isolates in IRBLsh-S, 91 isolates in IRBLz5-CA, 91 isolates in IRBLzt-T, and 84 isolates in LTH.

There were 10 significant SNPs on chromosomes 1, 2, 3, 4, 5 and 7 for IRBLsh-S, 8 SNPs on chromosomes 1, 2, 3, 5, and 6 for IRBLz5-CA, 5 SNPs on chromosomes 1, 2, and 6 for IRBLk-KA, 1 SNP on chromosome 3 for IRBLzt-T, 1 SNP each on chromosome 6 for IRBLb-B and LTH (Table 1 and Figure 5).

SNP-trait association analysis was performed using TASSEL v.5 to identify pathogenicity genes. Disease reactions on six rice varieties carrying different blast resistance genes, IRBLzt-T (*Pizt*), IRBLsh-S (*Pish*), IRBLk-KA (*Pik*), IRBLb-B (*Pib*), Lijiangxintuangheigu (LTH) (Sck), and IRBLz5-CA (*Pi2*), were used in the analysis. SNPs associated with average disease score were detected on chromosomes 1, 2, 3, 4, 5, 6, and 7 using MLM analysis (Table 1). In addition, the upstream and downstream (±50 kb) of the GWAS significant SNP, based on the GFF annotation file of reference sequence isolate 70-15 version MG8 was analyzed. Many predicted genes were found in the target regions on chromosome 1 (209 genes), 2 (164 genes), 3 (135 genes), 4 (33 genes), 5 (55 genes), 6 (145 genes), and 7 (79 genes) on the six rice varieties. These genes included both protein coding and pseudo-genes (Table S4). Most of these genes are described as putative uncharacterized proteins related to avirulence signaling pathways. A gene (code MGG_05332) described as *P. oryzae* 70-15 1-phosphatidylinositol-4,5-bisphosphate phosphodiesterase delta at position MG3-5427965 to MG3-5431132 on chromosome 3 was found associated with loss of pathogenicity in IRBLz5-CA. Another position on chromosome 6 bounded by markers MG6-1000199 to MG6-1002969 encoding gene MGG_17623–a hypothetical gene in which the protein is characterized as Zn (2)-C6 fungal-type domain-containing protein. This gene functions to reduce virulence in IRBLz5-CA.

### 3.5. Haplotype Block and SNP Effect

From the GWAS results, 23 out of the 26 cut-off SNPs were identified in the LD block (Figure S1), while the other three were not in the LD block. For the trait IRBLzt-T, IRBLsh-S, IRBLk-KA, IRBLz5-CA, LTH, and IRBLb-B, the number of GWAS significant SNPs inside the block was 1, 8, 5, 8, 0, and 1, respectively. Moreover, there are SNPs that were identified in the blocks other than the significant GWAS SNPs.

In addition, 17 blocks out of 23 had distance of less than 1 k. As shown in the Figure S1b, there were five blocks for the trait IRBLsh-S with 0 kb distance, namely blocks number 42 and 47 in chromosome 2, and another three blocks in chromosomes 3, 5, and 7. In the same way, there were also one block in chromosome 3 for IRBLzt-T, one block in chromosome 6 for IRBLb-B, five blocks in chromosomes 1, 2, and 6 for IRBLk-KA, and another five blocks for IRBLz5-CA in chromosomes 1, 3, and 6 (Figure S1a–f). On the other hand, six blocks with more than 1 kb distance were found in two traits; IRBLsh-S and IRBLz5-CA. For IRBLsh-S, blocks of 3 kb, 70 kb, and 43 kb were identified in chromosomes 1, 2, and 4 (Figure S1b). For IRBLz5-CA, the block of 90 kb, 39 kb, and 1 kb were in chromosomes 2, 3, and 5, respectively (Figure S1d).

The 11 SNPs for IRBLshS, five SNPs for IRBLkKA, and eight SNPs for IRBLz5CA were used in the multiple regression analysis (Table S5), and four, three, and four SNPs for IRBLsh-S, IRBLk-KA, and IRBLz5-CA, respectively, were retained. Together with one SNP each for IRBLzt-T, LTH, and IRBLb-B, a total of 14 SNPs comprised the final set of SNPs. 

**Figure 6:**
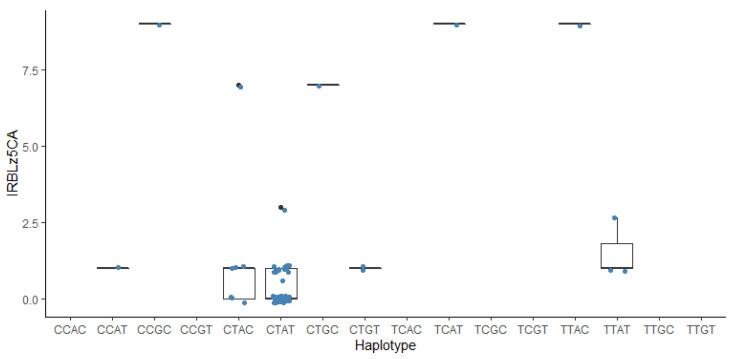
Haplotypes retained from multiple linear regression for the IRBLz5-CA. The first, second, third, and fourth allele in each combination were derived from MG2-5441644, MG3-1645410, MG6-703373, and MG6-957987 GWAS significant SNPs, respectively.

Haplotype analysis of four SNPs for IRBLz5-CA revealed that alleles C, T, A, and T from MG2_5441644, MG3_1645410, MG6_703373, and MG6_957987 contributed 18%, 28%, 14%, and 16% of PVE, which signals reduced virulence of the isolates against IRBLz5-CA carrying *Pi2* gene (Figure 6). Overall, the combination of alleles coming from four significant SNPs explained 69% of the phenotypic variation in damage scores of IRBLz5-CA. The allele combination CTAT showed the lowest damage score (0.37), while the allele combinations TCAT and TTAC had the highest damage score (9). The differences between low damage score from CTAT and high damage scores from TCAT (*t* (50) = 20.04, *p* = 0.001) and TTAC (*t* (50) = 20.04, *p* = 0.001) was statistically significant.

## 4. Discussion

The *M*. *oryzae* and *Oryza stiva* interaction has been shown to be a classic gene system [25], and a mutation in the avirulence gene (*Avr*) may be important for plant–pathogen interactions. In rice, the interaction between host plant and pathogen *M*. *oryzae* is well documented as a gene-for-gene system. In this study, we used a differential system consisting of five differential *R* genes and a universal susceptible LTH for the identification of the pathogenicity specific to neck blast resistance gene under greenhouse condition. Based on the disease score, IRBLzt-T and IRBLb-B–having *Pizt* and *Pib R* genes–and LTH were significantly susceptible to neck blast, and in rice variety F145-2 containing *Pi-b* gene showed that the resistance was broken down and caused leaf blast disease; thus, *Pi-b* gene may not be recommended in Thailand. The susceptibility of the *Pib* gene has been shown in the monogenic IRBLb-B as well as LTH when tested in three field blast nurseries in Korea [26] and in intermediate reactions for Bangladesh isolates [27]. Among the differential *R* genes, *Pish*, *Pik,* and *Pi2* showed strong resistance to leaf blast diseases not only in the greenhouse but also under field conditions [28,29]. For this reason, IRBLsh-S, IRBLk-KA, and IRBLz5-CA varieties carrying *Pish*, *Pik,* and *Pi2* or *Piz5 R* genes showed resistance against neck blast in many rice growing regions using different isolates. Therefore, these varieties can be useful to help breeders to select resistant *R* genes for breeding programs in Thailand. In a previous study, *R* genes (*Pik* (*Pikh*, *Pikm*, *Pi7* (*t*)), *Pi9*, *Pish*, *Pib*, *Piz*-*5* or *Pi2*, *Pita*, *Pita*-*2,* and *Pi19*) were recommended to be chosen for developing rice potentially resistant to blast disease breeding programs in Thailand [30]. *Pi1*, *Piz*-*5*, and *Pita*-*2* genes have been reported to be effectively resistant to blast disease in Thailand [31]. These identified genes not only provide new genetic resources for breeding broad-spectrum and durable rice varieties, but also new strategies to improve resistance to rice blast. To our knowledge, this was the first identification of blast pathogenicity genes specific to neck blast disease resistance genes identified by a genome-wide association study. 

The blast isolate population of *M*. *oryzae* can be divided into four groups, as shown in Figure 3. All clusters showed a genetic distribution indicating diverse genotypes. Each cluster contained different isolates coming from different regions of Thailand. The cluster analysis showed that most isolates collected at different times and regions–but mostly from the northeast–were grouped in clusters G3 and G4, indicating similarity in genotype of blast fungus found in that region of Thailand. IRBLkKA and IRBLshS showed 92% resistance against northeast isolates in G3 and G4 and 100% resistance in IRBlz5CA for the same group. The results indicate that resistance genes *Pik, Pish,* and *Pi2* maybe suitable in the northeast region of Thailand. On the other hand, *Pib* and *Pizt* may not show broad resistance in the northeast [32], as it has been demonstrated that resistance genes in the Thai rice variety Jao Hom Nin consists of *Pish* and *Pi7*. Jao Hom Nin had been used in breeding programs in Thailand by introgressing QTL for blast resistance found in Jao Hom Nin to the rice cultivar RD6 [33]. Unfortunately, the resistance genes in IRBLbB and IRBLztT resulted in only 46% and 83% resistance, respectively, against the northeast isolates in G3, and 72% and 33% resistance in IRBLbB and IRBLztT, respectively, against the northeast isolates in G4. This shows that resistance genes *Pib* and *Pizt* may not show broad resistance to blast in northeastern Thailand. Although there is a tendency for the blast isolates from the northeast to be grouped in G3 and G4, the rest of the isolates were mixed in every group and therefore cannot be distinguished by regional origin indicating low relationship between year of collection, location, and genotypic data. However, more investigation is needed to gain more information in the mixture of isolates in clusters G1 and G2. Thailand is known as an origin and place of high diversity of *M*. *oryzae* [34]. However, *M*. *Oryzae,* requires two mating types: MAT1-1 and MAT1-2. Additionally, it was observed in north, northeast, and central Thailand that most of the isolates were of the same mating type (MAT1-2) [34,35]. 

In this study, we aimed to identify SNPs associated with pathogenicity genes in *M*. *oryzae* that correspond to resistance genes for neck blast disease in six rice cultivars, by analyzing the associations between genetic variation of 102 isolates and their disease response on rice varieties. The analysis was performed with IRBLzt-T, IRBLsh-S, IRBLk-KA, IRBLb-B, IRBLz5-CA, and LTH (universal susceptible, genetic background of IRBL). A number of SNPs (5338) was used in genome wide association, and we found the significant SNPs linked to *Avr* genes on chromosomes 1, 2, 3, 4, 5, 6, and 7. Some of the chromosomes corresponded to leaf blast QTL on chromosomes 1, 2, 3, 4, and 7 [12], except for chromosomes 5 and 6, which were only detected for neck blast QTL. According to GWAS results, different varieties yielded different positions. 

The gene MGG_05332 has phosphatidylinositol phospholipase C activity related with intracellular signal transduction. This gene was found between MG3-5427965 to MG3-5431132 on chromosome 3, and about 29 Kb away from the GWAS significant SNP at position 5461007 for the variety IRBLz5-CA. From the Phi database (Phi-base.org), gene MGG_05332 is also known as *MoPLC2* (Phi ID: 2018) in which the mutant phenotype shows loss of pathogenicity. The C2 domain in *MoPLC2* is involved in Ca2+-dependent membrane binding. The hyphal growth rate of *MoPLC2* mutant was normal other than the conidial production being reduced, which probably explains the loss of pathogenicity [36]. This complement resistance was found in IRBLz5-CA, posting 81% resistance against 91 isolates tested. Another gene–MGG_17623–that was found 42 Kb from MG6-957987, the GWAS significant SNP, and bound by markers MG6-1000199 to MG6-1002969, revealed that in the Phi database (Phi ID:5661), the gene functions as a *Zn2Cys6* transcription factor and is responsible for reduced virulence in mutant phenotype. The *Zn2Cys6* transcription factor in *M*. *oryzae* is essential in fungal development, and is involved in pathogenicity at multiple developmental stages [37]. This study is the first to identify the avirulence gene specific for neck blast based on an association study in *M*. *oryzae*. The information from this study will be useful to narrow down and develop specific primers in the future. 

For haplotypes in relation to phenotype, there is lack of samples for some haplotypes, such as in IRBLsh-S, for which some allele combinations (AACT, AATC, AATT, AGCC, etc.) were not identified. By using more varieties and isolates, the separation of the phenotype from its haplotypes should be guaranteed. It was determined from Figure S2a that the allele C of the MG3_322529 results in avirulence of the fungus to IRBLzt-T variety. Meanwhile, the haplotype GG of MG2_7171094 and MG2_2428572 results in avirulence for IRBLsh-S. The haplotype CT of MG1_425230 and MG1_5639396 is an avirulence allele combination for IRBLk-KA (Figure S2c). For IRBLz5-CA, there are three haplotypes showing fungal avirulence reaction: T/A of MG3_1645410 and MG6_703373, C/T of MG2_5441644 and MG6_957987, and C/A of MG2_5441644 and MG6_703373 (Figure 5). Haplotype analysis has been used in various studies in rice to identify associations between patterns of genomic variants and phenotypes, such as for salt tolerance [38], grain thickness [39], and aroma [40,41,42]. In particular, haplotype analysis has been successfully utilized in marker development in rice disease resistance, such as for bacterial blight resistance [43]. For blast resistance, many studies determined haplotypes on *R* genes to identify resistance and susceptible haplotypes [44,45,46], which are also called functional and nonfunctional haplotypes [47]. In this study, haplotype analysis of six rice varieties suggests that each variety requires a specific marker for each corresponding avirulence gene. Specific markers can be subsequently used for identifying natural blast fungus that will be useful to select corresponding resistance genes in breeding programs and cultivation. 

## 5. Conclusions

Neck blast screening in this study revealed that *R* genes, *Pik*, *Pish,* and *Pi2* were broad spectrum resistance against 90% of Thai blast isolates inoculated. Moreover, these genes were the most resistant when *M*. *oryzae* isolates were grouped based on the genotypic data. This is the first gene identification study for pathogenicity in *M. oryzae* for neck blast disease on rice. GWAS and haplotype analyses resulted in the identification of 27 significant SNP positions on chromosomes 1 to 7 associated with pathogenicity of blast disease fungus. Haplotype analysis revealed that specific allele combinations reduced fungus virulence and therefore resulted in increasing blast resistance in rice. Two genes, MGG_05332 and MGG_17623, were reported to be related to virulence in *M. oryzae* and located close to significant SNPs in IRBLz5-CA, MG3-5461007, and MG6-957987 on chromosomes 3 and 6, respectively. Therefore, these genes should be useful for rice blast disease resistance breeding programs.

## Figures and Tables

**Table 1:** The position of significantly associated SNPs from association analysis of pathogenicity loci region in 102 isolates of *M. oryzae* against six rice varieties, using mixed linear model (MLM) on TASSEL software.

Trait	Chr	Marker	Position	SNP	LOD	*p* Value	R2 Marker
IRBLzt-T	3	MG3-322529	322529	T/C	3.120463	0.000758	0.14933
IRBlsh-S	1	MG1-7553101	7553101	G/T	3.917897116	0.000121	0.1842
	1	MG1-5731374	5731374	G/A	3.74562113	0.00018	0.17485
	1	MG1-7876964	7876964	T/C	3.026987666	0.00094	0.1347
	2	MG2-7171094	7171094	G/A	3.882529836	0.000131	0.18385
	2	MG2-2428572	2428572	G/A	3.610975749	0.000245	0.23869
	2	MG2-2406970	2406970	G/A	3.258139606	0.000552	0.17476
	3	MG3-254639	254639	T/C	3.051924624	0.000887	0.13761
	4	MG4-586828	586828	T/C	3.372071155	0.000425	0.15561
	5	MG5-4455619	4455619	C/T	3.147904454	0.000711	0.14121
	7	MG7-2400656	2400656	G/A	3.172818186	0.000672	0.14253
IRBLk-KA	1	MG1-425230	425230	C/T	3.865313007	0.000136	0.18733
	1	MG1-6669832	6669832	C/T	3.738594373	0.000183	0.15984
	1	MG1-5639396	5639396	T/G	3.107332284	0.000781	0.12795
	2	MG2-8137518	8137518	T/C	3.075736225	0.00084	0.12698
	6	MG6-1316128	1316128	T/C	3.050697837	0.00089	0.12485
IRBLb-B	6	MG6-3022354	3022354	A/G	3.359916269	0.000437	0.20947
LTH	6	MG6-1068242	1068242	G/C	3.305053	0.000495	0.20179
IRBLz5-CA	1	MG1-1250754	1250754	G/A	3.150543731	0.000707	0.14578
	2	MG2-5441644	5441644	C/T	3.797975105	0.000159	0.18779
	3	MG3-1645410	1645410	T/C	4.980800599	0.0000105	0.28816
	3	MG3-6316407	6316407	C/T	4.266963317	0.0000541	0.21868
	3	MG3-5461007	5461007	A/G	3.17157858	0.000674	0.14592
	5	MG5-54924	54924	C/T	3.588363461	0.000258	0.16954
	6	MG6-957987	957987	T/C	3.605916224	0.000248	0.17213
	6	MG6-703373	703373	A/G	3.185559279	0.000652	0.14687

## Data Availability

Data are contained within the article.

## Supplementary Materials

The following supporting information can be downloaded at: https://www.mdpi.com/article/10.3390/genes13050916/s1, Figure S1: LD blocks of cut-off SNPs for trait (a) IRBLzt-T, (b) IRBLsh-S, (c) IRBLk-KA, (d) IRBLz5-CA, (e) LTH, and (f) IRBLb-B. Yellow box indicates the cut-off SNP from GWAS and the black triangle indicates the LD block. The size of LD block is indicated in square bracket next to the block number. Above the black triangle is the id of SNP. The number inside the black triangle represents the ordering number of SNP in chromosome and the number in red, grey and white square represents r^2^; Figure S2: Haplotypes after analyzing multiple linear regression for the trait (a) IRBLzt-T, (b) IRBLsh-S, (c) IRBLk-KA, (d) LTH, (e) IRBLb-B; Table S1: Number of isolates and disease scores in six rice varieties; Table S2: Disease reaction data for six rice varieties of individual blast fungal isolate; Table S3. Linkage disequilibrium decay; Table S4. Gene annotations; Table S5. SNP IDs after passing each analyzing process; Table S6. One-hundred and two isolates of *M. oryzae* collected from several locations in Thailand during year 2001–2017.
