# Supplementary material for: Identification of Pathogenicity Loci in Magnaporthe oryzae Using GWAS with Neck Blast Phenotypic Data
Source: Genes (Basel). 2022 May 20;13(5):916. doi: 10.3390/genes13050916 (PMC9141631; doi:10.3390/genes13050916)
Supplement: Supplementary file 1 [file genes-13-00916-s001.zip › Supplementary Figures_Nyein.pdf]

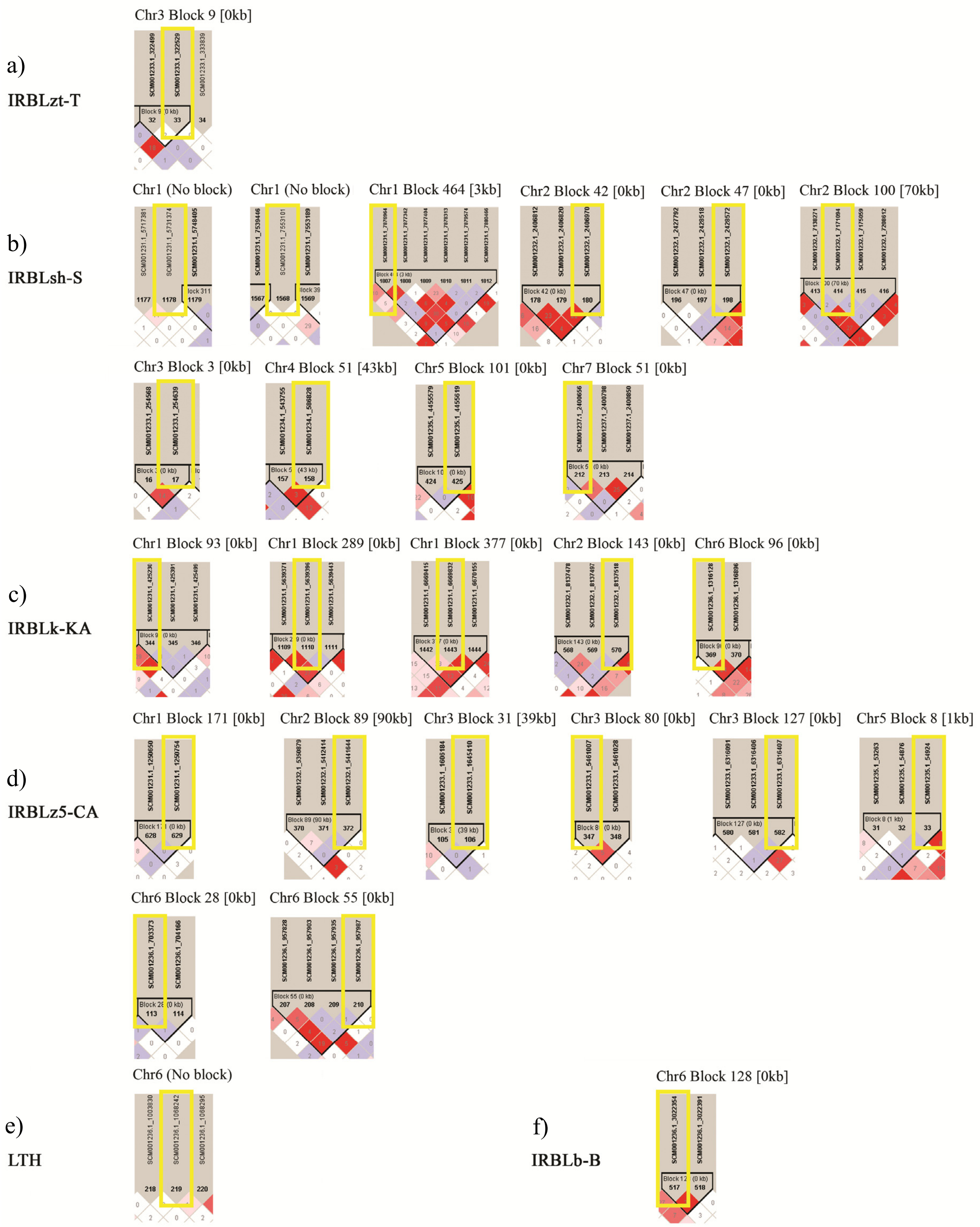

Figure S1. LD blocks of cut-off SNPs for trait (a) IRBLzt-T, (b) IRBLsh-S, (c) IRBLk-KA, (d) IRBLz5-CA, (e) LTH, and (f) IRBLb-B. Yellow box indicates the cut-off SNP from GWAS and the black triangle indicates the LD block. The size of LD block is indicated in square bracket next to the block number. Above the black triangle is the id of SNP. The number inside the black triangle represents the ordering number of SNP in chromosome and the number in red, grey and white square represents r^2.

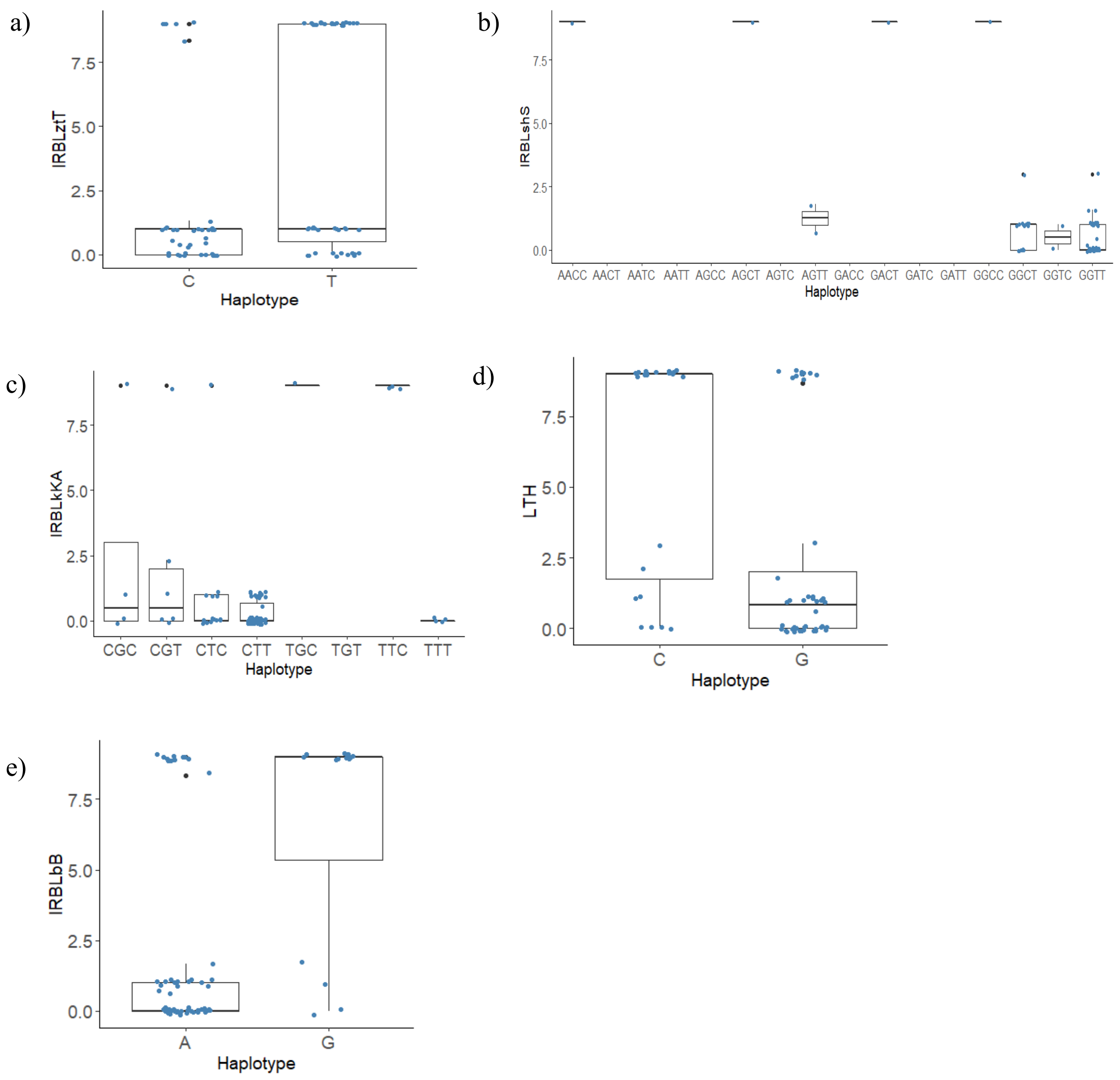

Figure S2. Haplotypes after analyzing multiple linear regression for the trait (a) IRBLzt-T, (b) IRBLsh-S, (c) IRBLk-KA, (d) LTH, (e) IRBLb-B.
