# Supplementary material for: Identification of Pathogenicity Loci in Magnaporthe oryzae Using GWAS with Neck Blast Phenotypic Data
Source: Genes (Basel). 2022 May 20;13(5):916. doi: 10.3390/genes13050916 (PMC9141631; doi:10.3390/genes13050916)
Supplement: Supplementary file 1 [file genes-13-00916-s001.zip › Supplementary Tables_Nyein.pdf]

Table S1. Number of isolates and disease scores in six rice varieties

Phenotype	IRBLztT DS	IRBlshS DS	IRBLkKA DS	IRBLbB DS	LTH DS	IRBLz5CA DS
Highly Resistant (HR)	26	43	65	38	30	53
Resistant (R)	37	39	21	18	19	30
Moderately Resistant (MR)	-	2	2	3	2	3
Moderately Susceptible (MS)	-	-	-	-	-	-
Susceptible (S)	1	-	-	-	-	3
Highly Susceptible (HS)	26	6	8	25	33	3
**Total**	**90**	**90**	**96**	**85**	**84**	**91**

Table S2. Disease reaction data for six rice varieties of individual blast fungal isolate

Isolates	Region	IRBLkKA	IRBLztT	IRBLshS	IRBlz5CA	LTH	IRBLbB
B1-2	Northeast	MR	HS	HS	HR	HR	R
BAG2-4	Northeast	HR	HS	R	HR	R	HS
BAG44-2	Northeast	HR	HR	HR	HR	R	HR
BKK55003	Central	R	R	HR	HR	R	S
BLAST4	North	HR	R	HR	-	HR	HR
BRM60002-1	Northeast	R	HS	HR	R	HS	HS
BRM60007-1	Northeast	R	HR	R	HR	HS	HS
BRM60009-1	Northeast	HR	HR	HR	R	MR	HS
CCO56003	East	HR	R	R	S	R	HS
CCO56004	East	R	HS	HR	HR	HR	HS
CRI59005	North	HR	HR	HR	HR	HS	HR
CRI60001-1	North	-	HR	R	-	-	-
CRI60003-2	North	HR	-	-	-	HR	HR
CRI60004-1	North	HR	R	HR	HR	HS	HR
KBI60001	South	HR	HR	R	HR	HR	HR
KKN61006	Northeast	HR	HS	HR	R	R	HS
LBR59001	Central	HR	HR	R	R	-	-
LRI59003-3	Central	HR	HS	MR	HR	R	HS
MKM61003	Northeast	HR	HS	R	HR	R	R
MSN60005-1	North	HR	HR	HR	HR	HS	HR
MSN60015	North	R	R	R	R	R	R
NBP60003-1	Northeast	HR	HS	R	R	R	R
NBP6001	Northeast	HR	HS	HR	HR	R	HR
NPM61001	Northeast	HR	HS	HR	R	HR	HS
NRT60001	South	-	-	-	R	HS	HS
NRT60002	South	MR	HS	HS	R	R	HS
PCT61003	Central	HR	R	HR	R	HS	HS
PL6007	South	HR	R	R	R	HS	HR

PNB59001-1	Central	HR	R	R	HS	HS	HS
PNB59003-3	Central	HS	HS	HS	R	HR	R
PRE59008-1	North	HR	HS	R	HR	R	HR
PRI57004	East	R	R	R	R	HS	R
PRI57014	East	HR	HS	HR	R	R	HS
RBR59001	West	-	R	R	HR	HR	HS
SIT61004	Central	HR	HR	HR	HR	R	HR
SKA60001	South	HR	R	HR	HR	HS	HR
SKA6002	South	HR	R	HR	HR	R	R
SKN60001-1	Northeast	R	R	R	HR	HS	HS
SKN60002-1	Northeast	HR	R	HS	HR	R	-
SKN60003	Northeast	R	HS	R	R	HR	R
SKW57001	East	HR	R	HR	-	-	-
SKW57006	East	R	-	HR	S	HS	HS
SKW57011	East	R	HS	R	HR	HR	HS
SPB60009	Central	HR	HR	HR	R	R	HS
SPB60012	Central	HR	HS	-	HR	R	-
SRN60001	Northeast	R	R	HR	R	HS	R
SRN60005-1	Northeast	HR	HR	R	MR	HS	HR
TH196031	Northeast	HR	R	R	HR	HS	HR
TH196036	Northeast	HS	HR	HR	HR	HR	HS
THL041	Central	HR	HS	HR	S	-	HR
THL060	North	HR	R	HS	S	HS	R
THL068	Central	R	HS	R	HR	HS	HR
THL1008	East	HR	-	HR	-	HR	HS
THL1109	South	HR	HR	R	HR	-	HR
THL112	Northeast	HS	HS	R	-	HR	HS
THL1126	South	R	R	R	HR	-	R
THL138	North	R	R	R	HR	HR	HR
THL143	North	HR	HR	HR	HR	-	HR

THL144	North	HR	HR	-	-	-	-
THL149	North	HR	HR	-	R	HS	HR
THL190	Central	HR	HS	R	HR	HR	R
THL208	Central	HR	R	R	MR	HS	-
THL234	Central	HR	S	-	R	-	HR
THL274	Central	HR	HS	R	R	HR	R
THL279	North	HR	R	R	R	HS	R
THL543-2	Central	HR	R	R	R	HR	R
THL652	North	HR	R	HR	HR	HS	R
THL727	Central	HR	R	R	R	HS	HR
THL831	North	HR	R	HR	HR	R	HR
THL881	South	-	R	R	HR	-	R
THL903	West	HR	R	R	R	HR	HS
THL934	Northeast	HR	HR	-	-	HR	-
THL943	Central	HS	-	HR	HR	MR	HS
THL959	South	HR	-	HR	HR	HS	HR
THL967	Northeast	HR	-	-	R	HS	HR
THL972	Northeast	HR	HR	HR	HR	HR	HR
THL983	Northeast	HR	HR	HR	HR	HR	R
THL992	Central	HR	R	HR	HS	HS	-
TRG1	Northeast	R	-	R	R	HS	HR
TRG10	North	HS	R	HR	R	HS	HR
TRG12	North	HS	HS	MR	HS	-	R
TRG15	North	HR	R	HR	HR	HR	HR
TRG17	North	HR	HR	HR	R	HR	HR
TRG2	Northeast	HS	HS	HS	HR	HR	HS
TRG20	Northeast	HR	R	HR	HR	HR	R
TRG26	North	R	HR	R	HR	HR	HR
TRG30	Central	R	HR	R	-	HR	HR
TRG34	Central	R	HR	HR	HR	HS	R

TRG36	Central	HR	R	HR	HR	HS	HR
TRG38	North	HR	HR	-	HR	HS	HR
TRG41	North	HR	-	HR	HR	-	-
TRG43	West	HR	R	HR	-	-	HR
TRG44	Central	-	-	-	HR	-	-
TRG47	Northeast	R	HR	HR	HR	HS	HR
TRG5	North	HR	HS	R	HR	HR	HR
TRG8	North	HS	R	R	-	-	HR
UDN60001.1	Northeast	-	-	-	HR	-	-
UDN60003-2	Northeast	R	HS	R	-	-	-
UTD17002	North	HR	-	-	-	-	HR
UTI61007	Central	HR	HR	HR	HR	HR	-
YST61004	Northeast	HR	R	HR	HR	HR	-
phitsanulok-40-4	Central	R	R	R	R	HS	R

Table S3. Linkage disequilibrium decay

Distance	Linkage Disequilibrium (r^2)							
(bp)	Mean	Chr1	Chr2	Chr3	Chr4	Chr5	Chr6	Chr7
10	0.7147	0.6877	0.7888	0.7299	0.6107	0.7392	0.6921	0.6847
20	0.6007	0.5728	0.6663	0.6423	0.5331	0.6163	0.5763	0.5318
30	0.5339	0.5112	0.5862	0.6021	0.4651	0.5496	0.4924	0.5023
40	0.4867	0.4615	0.5455	0.5543	0.4184	0.5037	0.4464	0.3926
50	0.4384	0.4181	0.5003	0.5307	0.3469	0.4402	0.3927	0.3240
60	0.4153	0.4087	0.4679	0.5269	0.3276	0.4124	0.3366	0.2953
70	0.3809	0.3679	0.4341	0.4844	0.3010	0.3776	0.3157	0.2671
80	0.3625	0.3474	0.4163	0.4908	0.2793	0.3493	0.2819	0.2376
90	0.3489	0.3328	0.4076	0.4828	0.2825	0.3339	0.2755	0.2100
100	0.3343	0.3149	0.3959	0.4560	0.2929	0.3237	0.2560	0.2322
30000	0.1092	0.0874	0.1758	0.1534	0.0911	0.1022	0.0653	0.0998
60000	0.0539	0.0395	0.0889	0.0600	0.0441	0.0323	0.0413	0.0719
90000	0.0464	0.0471	0.0654	0.0301	0.0754	0.0252	0.0441	0.0611
120000	0.0487	0.0488	0.0472	0.0509	0.0480	0.0418	0.0495	0.0682
150000	0.0570	0.0611	0.0419	0.0524	0.0645	0.0402	0.0562	0.0981
180000	0.0457	0.0438	0.0585	0.0515	0.0693	0.0611	0.0427	0.0870
210000	0.0401	0.0351	0.0533	0.0514	0.0501	0.0516	0.0494	0.0719
240000	0.0483	0.0471	0.0557	0.0446	0.0679	0.0478	0.0486	0.0430
270000	0.0489	0.0468	0.0462	0.0540	0.0474	0.0705	0.0462	0.0835
300000	0.0521	0.0416	0.0591	0.0548	0.0729	0.0730	0.0628	0.0750
330000	0.0445	0.0323	0.0656	0.0629	0.0564	0.0932	0.0559	0.0882
360000	0.0426	0.0319	0.0671	0.0591	0.0670	0.1094	0.0479	0.0855
390000	0.0455	0.0372	0.0722	0.0541	0.0566	0.0794	0.0488	0.0723
420000	0.0461	0.0429	0.0457	0.0425	0.0853	0.0723	0.0515	0.0813
450000	0.0438	0.0368	0.0678	0.0399	0.0779	0.0645	0.0535	0.0620
480000	0.0430	0.0342	0.0560	0.0391	0.0554	0.0693	0.0549	0.0927
510000	0.0380	0.0353	0.0305	0.0365	0.0779	0.0601	0.0566	0.0496
540000	0.0462	0.0419	0.0400	0.0665	0.0463	0.0752	0.0613	0.0569
570000	0.0404	0.0448	0.0294	0.0450	0.0507	0.0582	0.0380	0.0996
600000	0.0350	0.0300	0.0355	0.0528	0.0527	0.0488	0.0380	0.0901
630000	0.0309	0.0285	0.0273	0.0468	0.0630	0.0341	0.0441	0.0949
660000	0.0310	0.0287	0.0287	0.0475	0.0508	0.0270	0.0428	0.0875
690000	0.0385	0.0354	0.0347	0.0438	0.0549	0.0512	0.0619	0.0684
720000	0.0442	0.0368	0.0455	0.0719	0.0677	0.0391	0.0479	0.0787
750000	0.0550	0.0490	0.0575	0.0862	0.0761	0.0510	0.0504	0.1039
780000	0.0461	0.0348	0.0809	0.0733	0.0831	0.0466	0.0443	0.0723
810000	0.0396	0.0310	0.0819	0.0729	0.0598	0.0669	0.0417	0.1061
840000	0.0442	0.0345	0.0957	0.0727	0.0774	0.0289	0.0514	0.1066
870000	0.0434	0.0325	0.0838	0.0597	0.0788	0.0358	0.0627	0.1025
900000	0.0421	0.0367	0.0831	0.0921	0.0776	0.0258	0.0752	0.0680
930000	0.0529	0.0374	0.0892	0.0892	0.0649	0.0547	0.0842	0.1124
960000	0.0451	0.0312	0.0949	0.0687	0.0544	0.1861	0.0745	0.0624
990000	0.0395	0.0290	0.0862	0.0701	0.0528	0.0967	0.0616	0.0535
1020000	0.0363	0.0280	0.0792	0.0725	0.0632	0.1082	0.0750	0.0624

Table S5. SNP IDs after passing each analyzing process

Trait	Block multi. regression	Trait multi. regression
IRBLztT	MG3-322529	MG3-322529 (12%)
IRBLshS	MG1-7553101	
	MG2-7171094	MG2-7171094 (18%)
	MG1-5731374	
	MG2-2428572	MG2-2428572 (23%)
	MG2-2428518	
	MG2-2406970	
	MG1-7876964	
	MG3-254639	MG3-254639 (13%)
	MG4-586828	MG4-586828 (15%)
	MG5-4455619	
	MG7-2400798	
IRBLkKA	MG1-425230	MG1-425230 (21%)
	MG1-6669832	
	MG1-5639396	MG1-5639396 (12%)
	MG2-8137518	
	MG6-1316128	MG6-1316128 (12%)
IRBLz5CA	MG3-1645410	MG3-1645410 (28%)
	MG3-6316407	
	MG2-5441644	MG2-5441644 (18%)
	MG5-54924	
	MG6-957987	MG6-957987 (16%)
	MG6-703373	MG6-703373 (14%)
	MG3-5461007	
	MG1-1250754	
LTH	MG6-1068242	MG6-1068242 (19%)
IRBLbB	MG6-3022354	MG6-3022354 (21%)

Table S6. One-hundred and two isolates of *M. oryzae* collected from several locations in Thailand during year 2001-2017

No.	Isolate	Region in Thailand	Province	Collected year	Source
1	B1-2	Northeast	Ubon Ratchathani	2001	BIOTEC
2	BAG2.4	Northeast	Ubon Ratchathani	2016	Kasetsart University
3	BAG44.2	Northeast	Roi Et	2016	Kasetsart University
4	BKK55003	Central	Bangkok	2012	KMITL
5	BLAST4	North	Lampang	2014	RMUTL
6	BRM60002.1	Northeast	Buri Ram	2017	KMITL
7	BRM60007.1	Northeast	Buri Ram	2017	KMITL
8	BRM60009.1	Northeast	Buri Ram	2017	KMITL
9	CCO56003	East	Chachoengsao	2012	KMITL
10	CCO56004	East	Chachoengsao	2013	KMITL
11	CRI59005	North	Chiang Rai	2016	KMITL
12	CRI60001.1	North	Chiang Rai	2017	KMITL
13	CRI60003.2	North	Chiang Rai	2017	KMITL
14	CRI60004.1	North	Chiang Rai	2017	KMITL
15	KBI60001	South	Krabi	2017	KMITL
16	KKN61006	Northeast	Khon Kaen	2018	KMITL
17	LBR59001	Central	Lopburi	2016	KMITL
18	LRI59003.3	Central	Lopburi	2016	KMITL
19	MKM61003	Northeast	Mahasarakham	2018	KMITL
20	MSN60005.1	North	Mae Hong Son	2017	KMITL
21	MSN60015	North	Mae Hong Son	2017	KMITL
22	NBP60001	Northeast	Nong Bua Lam Phu	2017	KMITL
23	NBP60003.1	Northeast	Nong Bua Lam Phu	2017	KMITL
24	NPM61001	Northeast	นครพนม	2018	KMITL
25	NRT60001	South	Nakhon Si Thammarat	2017	KMITL
26	NRT60002	South	Nakhon Si Thammarat	2017	KMITL
27	PCT61003	Central	Phichit	2018	KMITL
28	PL60007	South	Phatthalung	2017	KMITL
29	PNB59001.1	Central	Phetchabun	2016	KMITL
30	PNB59003.3	Central	Phetchabun	2016	KMITL
31	PRE59008.1	North	Phrae	2016	KMITL
32	PRI57004	East	Prachin Buri	2014	KMITL
33	PRI57014	East	Prachin Buri	2014	KMITL
34	RBR59001	West	Ratchaburi	2016	KMITL
35	SKA60001	South	Songkhla	2017	KMITL
36	SKA60002	South	Songkhla	2017	KMITL

37	SKN60001.1	Northeast	Sakon Nakhon	2017	KMITL
38	SKN60002.1	Northeast	Sakon Nakhon	2017	KMITL
39	SKN60003	Northeast	Sakon Nakhon	2017	KMITL
40	SKW57001	East	Sa Kaeo	2014	KMITL
41	SKW57006	East	Sa Kaeo	2014	KMITL
42	SKW57011	East	Sa Kaeo	2014	KMITL
43	SPB60009	Central	Suphan Buri	2017	KMITL
44	SPB60012	Central	Suphan Buri	2017	KMITL
45	SRN60001	Northeast	Surin	2017	KMITL
46	SRN60005.1	Northeast	Surin	2017	KMITL
47	STI61004	Central	Sukhothai	2018	KMITL
48	TH196031	Northeast	Ubon Ratchathani	2001	Ubon Ratchathani rice research center
49	TH196036	Northeast	Ubon Ratchathani	2001	Ubon Ratchathani rice research center
50	THL041	Central	Phitsanulok	2001	BIOTEC
51	THL060	North	Chiang Mai	2001	BIOTEC
52	THL068	Central	Phitsanulok	2001	BIOTEC
53	THL112	Northeast	Surin	2001	BIOTEC
54	THL138	North	Chiang Mai	2001	BIOTEC
55	THL143	North	Chiang Mai	2001	BIOTEC
56	THL144	North	Chiang Mai	2001	BIOTEC
57	THL149	North	Chiang Mai	2001	BIOTEC
58	THL190	Central	Phitsanulok	2001	BIOTEC
59	THL208	Central	Phitsanulok	2001	BIOTEC
60	THL234	Central	Pathum Thani	2001	BIOTEC
61	THL274	Central	Chai Nat	2001	BIOTEC
62	THL279	North	Phrae	2001	BIOTEC
63	THL543-2	Central	Phetchabun	2001	BIOTEC
64	THL652	North	Chiang Mai	2001	BIOTEC
65	THL727	Central	Pathum Thani	2001	BIOTEC
66	THL831	North	Mae Hong Son	2001	BIOTEC
67	THL881	South	Chumphon	2001	BIOTEC
68	THL903	West	Ratchaburi	2001	BIOTEC
69	THL934	Northeast	Nong Khai	2001	BIOTEC
70	THL943	Central	Phra Nakhon Si Ayutthaya	2001	BIOTEC
71	THL959	South	Phatthalung	2001	BIOTEC
72	THL967	Northeast	Surin	2001	BIOTEC
73	THL972	Northeast	Khon Kaen	2001	BIOTEC
74	THL983	Northeast	Nong Khai	2001	BIOTEC

75	THL992	Central	Pathum Thani	2001	BIOTEC
76	THL1008	East	Trat	2001	BIOTEC
77	THL1109	South	Krabi	2001	BIOTEC
78	THL1126	South	Phuket	2001	BIOTEC
79	TRG1	Northeast	Nong Khai	2013	BIOTEC
80	TRG2	Northeast	Nong Khai	2013	BIOTEC
81	TRG5	North	Lampang	2014	BIOTEC
82	TRG8	North	Nan	2014	BIOTEC
83	TRG10	North	Nan	2014	BIOTEC
84	TRG12	North	Nan	2014	BIOTEC
85	TRG15	North	Lampang	2014	BIOTEC
86	TRG17	North	Lampang	2014	BIOTEC
87	TRG20	Northeast	Sakon Nakhon	2558	BIOTEC
88	TRG26	North	Phayao	2558	BIOTEC
89	TRG30	Central	Nakhon Pathom	2016	BIOTEC
90	TRG34	Central	Nakhon Pathom	2016	BIOTEC
91	TRG36	Central	Nakhon Pathom	2016	BIOTEC
92	TRG38	North	Phayao	2016	BIOTEC
93	TRG41	North	Chiang Rai	2017	BIOTEC
94	TRG43	West	Phetchaburi	2018	BIOTEC
95	TRG44	Central	Phra Nakhon Si Ayutthaya	2018	BIOTEC
96	TRG47	Northeast	Nong Bua Lam Phu	2018	BIOTEC
97	UDN60001.1	Northeast	Udon Thani	2017	KMITL
98	UDN60003.2	Northeast	Udon Thani	2017	KMITL
99	UTI61007	Central	Uthai Thani	2018	KMITL
100	YST61004	Northeast	Yasothon	2018	KMITL
101	พิษณุโลก40.4	Central	Phitsanulok	2016	KMITL
102	UTD17002	North	Uttaradit	2017	KMITL
